# Supplementary material for: Decoding Local Adaptation in the Exploited Native Marine Mussel Mytilus chilensis: Genomic Evidence from a Reciprocal Transplant Experiment
Source: Int J Mol Sci. 2025 Jan 23;26(3):931. doi: 10.3390/ijms26030931 (PMC11817969; doi:10.3390/ijms26030931)
Supplement: Supplementary file 1 [file ijms-26-00931-s001.zip › Data S1 RNA-Seq vs. qRT-PCR comparison.pdf]

TPM values vs. Relative expression comparison and validation.

TPM values were compared to relative expression values by qRT-PCR for some differentially expressed genes (DEGs), with β-actin used as the internal reference gene. Gene expression levels were calculated using the $2^{-\Delta\Delta Ct}$ method (Livak and Schmittgen 2001)[1]. Statistical analysis was performed using ANOVA, applying a multiple linear regression model in R v4.3. The model was tested for linearity, homoscedasticity, and normality using the Ramsey, Levene, and Shapiro tests. Results and conclusion are presented along with the following supplementary figures, scripts, and additional information.

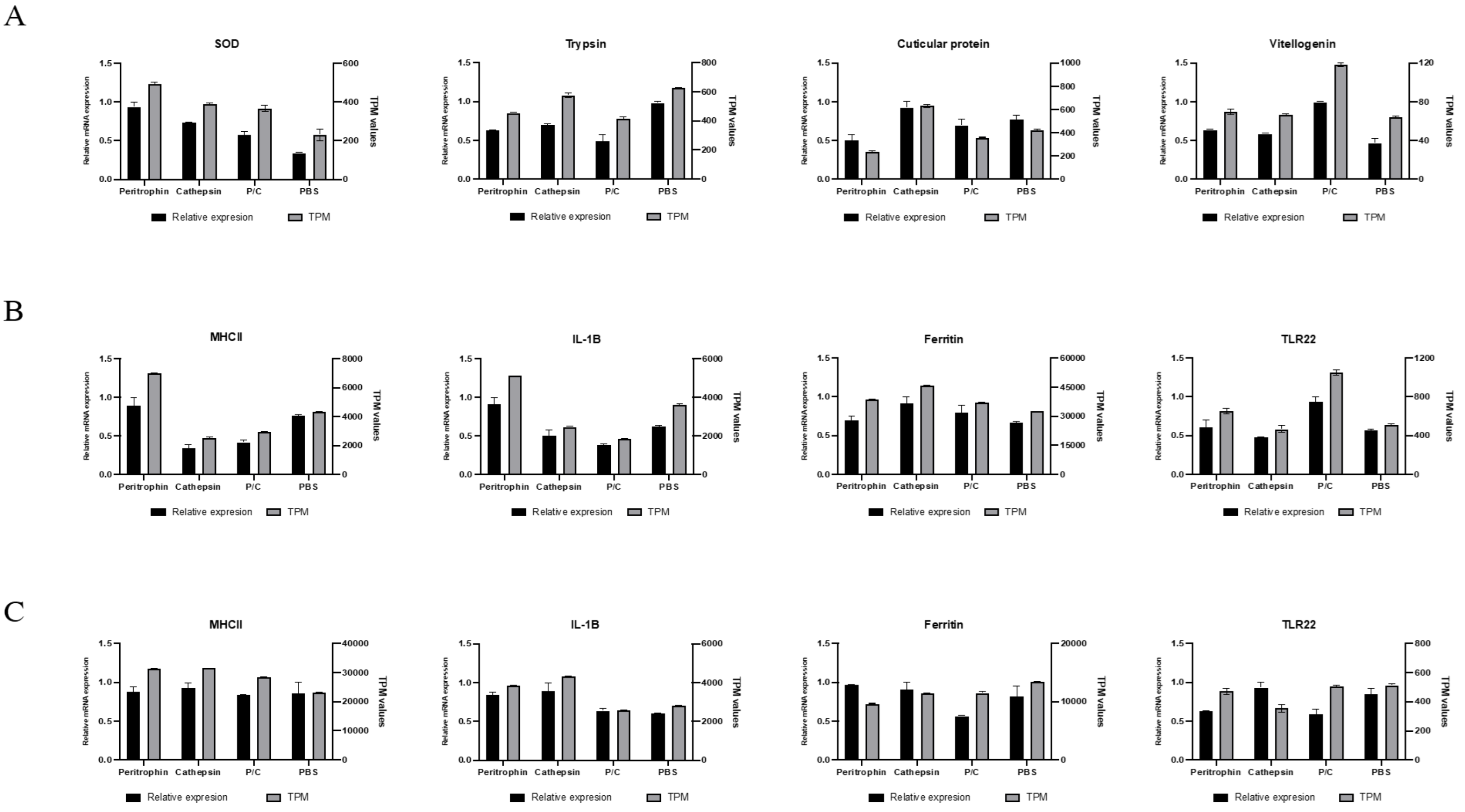

Lineal model:

```
> lm1<-lm(RelativExpress~TPM+Gene, data=datalab)
```

Residual vs. predicted values for model

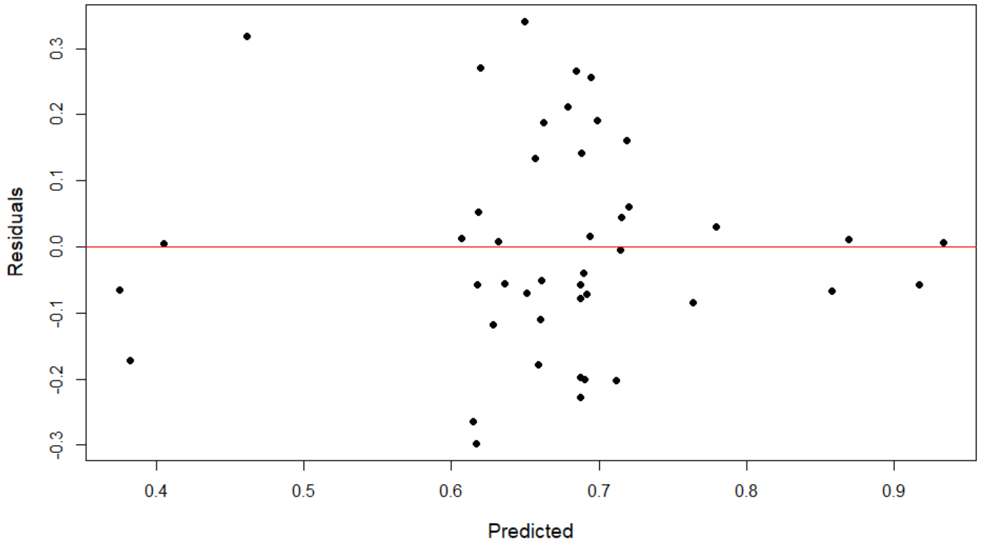

> Anova(lm1)
Anova Table (Type II tests)

Response: RelativExpress

	Sum Sq	Df	F value	Pr(>F)
TPM	0.05619	1	1.3673	0.2494
Gene	0.05128	7	0.1783	0.9883
Residuals	1.60271	39		

> summary(lm1)

Call:
lm(formula = RelativExpress ~ TPM + Gene, data = datalab)

Residuals:

Min	1Q	Median	3Q	Max
-0.40348	-0.11416	-0.01019	0.15775	0.33326

Coefficients:

	Estimate	Std. Error	t value	Pr(>|t|)
(Intercept)	7.142e-01	1.014e-01	7.047	1.85e-08 ***
TPM	1.856e-06	1.587e-06	1.169	0.249
GeneFeritin	-9.214e-02	1.768e-01	-0.521	0.605
GeneIL-B1	-7.538e-02	1.242e-01	-0.607	0.547
GeneMHCII	-1.061e-01	1.267e-01	-0.837	0.408
GeneSOD	-9.742e-02	1.433e-01	-0.680	0.501
GeneTLR22	-2.654e-02	1.241e-01	-0.214	0.832
GeneTrypsin	-2.270e-02	1.433e-01	-0.158	0.875
GeneVitellogenin	-5.773e-02	1.433e-01	-0.403	0.689

Signif. codes: 0 '***' 0.001 '**' 0.01 '*' 0.05 '.' 0.1 ' ' 1

Residual standard error: 0.2027 on 39 degrees of freedom
Multiple R-squared: 0.09496, Adjusted R-squared: -0.09068
F-statistic: 0.5115 on 8 and 39 DF, p-value: 0.8403

```
> #==================================================================
> #  Test de Tukey
> glht1<-glht(lm1,linfct=mcp(Gene="Tukey"))
> cld(glht1)
CuticularProt      Feritin        IL-B1        MHCII         SOD
         "a"          "a"          "a"          "a"          "a"

TLR22       Trypsin  Vitellogenin
"a"          "a"          "a"
```

Conclusion:
There are no significant differences between the gene expression values estimated through TMP (RNA-Seq) and relative expression using qRT-PCR.

Reference:

1. Livak, K.J.; Schmittgen, T.D. Analysis of Relative Gene Expression Data Using Real-Time Quantitative PCR and the 2−ΔΔCT Method. *Methods* **2001**, *25*, 402–408, doi:10.1006/meth.2001.1262.
