# Supplementary figures and images for: Decoding Local Adaptation in the Exploited Native Marine Mussel Mytilus chilensis: Genomic Evidence from a Reciprocal Transplant Experiment
Source: Int J Mol Sci. 2025 Jan 23;26(3):931. doi: 10.3390/ijms26030931 (PMC11817969; doi:10.3390/ijms26030931)

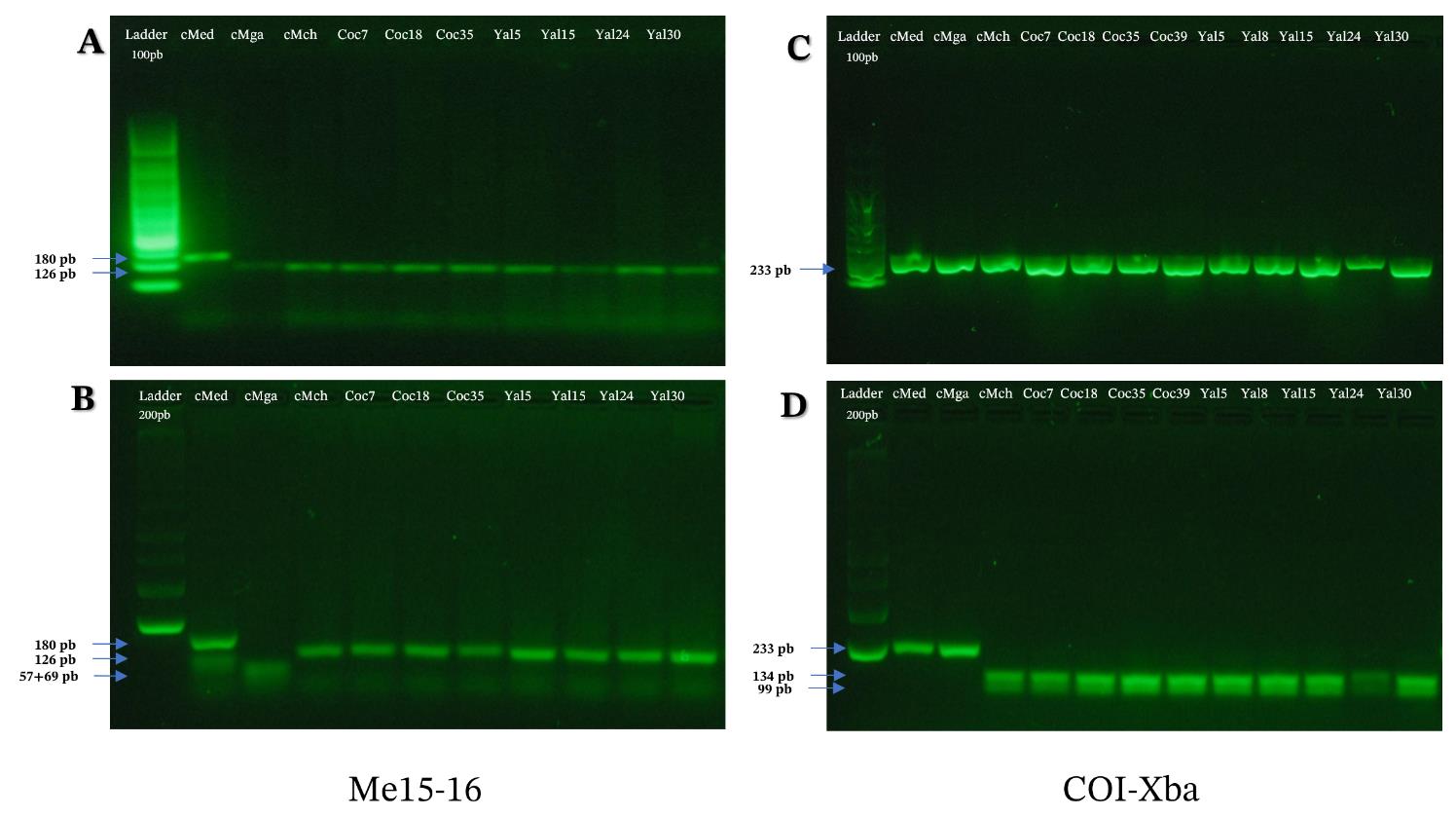

Supplement: Supplementary file 1 [file ijms-26-00931-s001.zip › Figure S1 Taxonomic affiliation.jpg]
